# The Effect of Foreign Language Perceptual Curiosity on Pragmatic Production Among Chinese EFL Junior and Senior Adolescent Students: A Moderated Mediation Model of Growth Language Mindset and Enjoyment

**DOI:** 10.3390/bs16101931

**Published:** 2026-10-17

**Authors:** Yuqi Wang, Sirui Chen

**Affiliations:** School of Foreign Languages, Beihang University, Beijing 100191, China; 13682632518@buaa.edu.cn

**Keywords:** pragmatic production, foreign language perceptual curiosity, enjoyment, growth language mindset, Chinese adolescent EFL learners

## Abstract

Pragmatic production requires the integration of pragmatic knowledge and socio-affective resources, yet the psychological mechanisms underlying productive pragmatic competence among adolescent EFL learners remain insufficiently explored. This study investigates how foreign language perceptual curiosity (FLPC) is associated with the pragmatic production among Chinese junior and senior secondary EFL students, focusing on the mediating role of foreign language enjoyment (FLE) and the moderating role of growth language mindset (GLM). Data were collected from 381 Chinese adolescent students through questionnaires and a discourse completion task. Structural equation modeling and moderated mediation analyses were conducted. Results showed that FLPC did not directly predict pragmatic production in either junior or senior groups. Instead, a statistical mediation pattern involving FLE was observed between FLPC and pragmatic production across both groups. Furthermore, GLM significantly moderated the relationship between FLPC and FLE among senior students, with the positive association being stronger at higher levels of GLM; however, this moderating effect was not observed among junior secondary students. These findings extend understanding of the affective and cognitive mechanisms underlying L2 pragmatic production by identifying an indirect association between situational curiosity and productive pragmatic performance through positive emotional engagement and a group-specific pattern in the role of growth-oriented learning beliefs.

## 1. Introduction

Pragmatic competence—the ability to use language appropriately in different social contexts—is a key part of second language competence (Taguchi & Roever, 2017). Compared to receptive modality, pragmatic production has dominated the field (Kasper & Rose, 2002) and is especially complex because it requires learners to not only understand social rules but also to manage the effects of their language on other participants and practice recipient design in real-time communication (Ren, 2022). Requests, as face-threatening acts (Brown & Levinson, 1987), have been the most extensively investigated speech act in pragmatic production (Taguchi & Roever, 2017). Meanwhile, existing research on L2 request production has primarily focused on adult populations, including university-level learners (Al-Gahtani & Roever, 2015), with only limited attention to learners of other age groups, including secondary and senior high school students (Rose, 2009), particularly under Chinese junior and senior EFL contexts. More importantly, existing assessment rubrics for assessing L2 productive pragmatic performances of requests have adopted holistic ratings (Taguchi, 2007), or focused on the distributional patterns of request strategies (Al-Gahtani & Roever, 2015; Savić et al., 2021), with few adopting a multi-dimensional, analytic scoring framework. Therefore, the present study employs the multi-layered pragmatic assessment rubric developed by Ishihara and Cohen (2022) to examine L2 request production between two groups of junior and senior high school EFL learners in China.

To date, research on individual difference (ID) variables affecting L2 pragmatic production has attracted great attention (Zhang & Aubrey, 2024; Taguchi & Roever, 2017), mainly encompassing L2 proficiency (Roever et al., 2014) and study-abroad experience (Ren, 2018), with occasional attention paid to motivational and personality variables, such as motivation (Yang & Wu, 2022) and personality (Taguchi, 2014). More recently, as positive psychology (PP) has gained increasing traction in L2 acquisition research (Dewaele & MacIntyre, 2014), the field of L2 pragmatics—recognized as a key component of overall L2 competence (Ren, 2022)—has gradually begun to examine a range of positive psychological variables, including emotions and L2 grit (Y. Wang & Ren, 2024), and language mindsets (Y. Wang & Ren, 2025; Zarrinabadi et al., 2021). However, these L2 pragmatic studies within the framework of positive psychology have largely focused on the modality of pragmatic reception, thereby leaving the association between PP variables and pragmatic production underexplored.

The model of factors influencing L2 pragmatics identifies socio-affective variables as a key component of individual learner characteristics alongside cognitive abilities and L2 proficiency (Ren, 2018). This framework also suggests that FLPC, conceptualized as a situational response to target language (TL) novelty rather than a stable personality trait, and distinct from epistemic curiosity (Henry et al., 2026), might serve as the situational affective variable, further influencing L2 pragmatic performances. Meanwhile, curiosity is treated as the construct that is “beneficial for learning and use of languages” (Henry et al., 2026, p. 3272). Considering that “L2 use is central to L2 pragmatics” (Y. Wang & Ren, 2025, p. 2296), it is legitimately plausible that the underlying linkage between FLPC and pragmatic production warrants empirical investigation. Emotional variables, including FLE and foreign language anxiety, have been examined as affective mediators in receptive pragmatic competence, with anxiety demonstrating a significant mediating role (Y. Wang & Ren, 2024). However, whether FLE specifically functions as a mediating mechanism within the domain of L2 pragmatic production of requests remains unaddressed. Furthermore, GLM has been associated with enjoyment (Hu et al., 2022; Yin et al., 2025), and has been shown to moderate the relationship between teacher support and L2 willingness to communicate (WTC) (Hejazi et al., 2023). Indeed, language mindset engages in complex, non-linear interactions with L2 emotional or motivational variables (Lou & Noels, 2017, 2019), thereby directly predicting L2 pragmatic awareness (Y. Wang & Ren, 2025). Nevertheless, its moderating role within the pathways connecting FLPC and L2 enjoyment to pragmatic production of requests remains unestablished. Drawing upon the framework of factors influencing L2 pragmatic competence (Ren, 2018), Control–Value Theory (CVT) (Pekrun, 2006), and the Language-Mindset Meaning System (LMMS) (Lou & Noels, 2019), this study investigates how FLPC is associated with pragmatic production of requests among Chinese EFL junior and senior learners, alongside the mediating role of FLE and GLM’s moderating path.

## 2. Literature Review and Hypotheses Development

### 2.1. Theoretical Frameworks

Ren (2018) proposed a model of factors influencing L2 pragmatic development, identifying three interrelated categories of individual learner characteristics: socio-affective variables (including attitudes, motivation, and emotions), cognitive abilities, and L2 proficiency. This framework underscores that pragmatic competence, particularly pragmatic production, emerges from the dynamic interplay of these multiple factors rather than from any single variable in isolation. Crucially, L2 pragmatic competence has been shown to proceed in a variable and non-linear manner shaped by the complex interaction of individual and contextual factors (Taguchi & Roever, 2017), suggesting that no single pathway sufficiently captures the mechanisms underlying pragmatic production. It is therefore necessary to examine multiple situational and affective variables simultaneously from a comprehensive perspective.

CVT (Pekrun, 2006) has proposed that learners’ appraisals of subjective control and value are central to the generation of achievement emotions in academic contexts. Within this framework, situational variables—such as FLPC in this study—function as distal antecedents that shape these appraisals and thereby trigger activity emotions, in this case, L2 enjoyment. CVT thus offers a solid basis for examining how such situational-emotional pathways may jointly contribute to academic achievement outcomes, that is, L2 pragmatic production of requests examined in the present study.

Building on the Language Mindsets Inventory (LMI; Lou and Noels (2017)), Lou and Noels (2019) proposed LMMS, positioning growth and fixed language mindsets as the central organizing construct of an integrated system in which mindsets are dynamically interconnected with motivational variables, self-regulation tendencies, and emotional experiences—rather than as isolated, linear relationships. This multidimensional framework highlights that the influence of mindsets and other individual differences on learning outcomes is context-sensitive and shaped by its interplay with affective and motivational factors (Ren, 2022; Yang & Ren, 2019). Empirical evidence has further linked growth language mindset to L2 pragmatic awareness among Chinese EFL learners (Y. Wang & Ren, 2025), suggesting its potential role in shaping L2 productive pragmatic performances.

### 2.2. Pragmatic Production

Within pragmatic production research, speech acts have been the primary focus (Ren, 2022). Studies on L2 pragmatic production have mainly examined requests (Savić et al., 2021), specifically among Chinese EFL learners (Tian & Li, 2026). Appropriate request performances require sensitivity to contextual variables of power, social distance, and degree of imposition (Brown & Levinson, 1987), and have consistently attracted the greatest volume of empirical attention (Ren, 2022). Requests are among the most frequently performed speech acts in daily communication (Taguchi & Roever, 2017) and are particularly relevant to adolescent EFL learners, whose classroom and social interactions regularly require this function (Alcón Soler, 2013). However, few studies have specifically examined the pragmatic production of requests among Chinese junior and senior secondary EFL learners.

A growing body of research has investigated motivational and PP variables in L2 pragmatics, yet the outcome measures consistently target receptive pragmatic competence. For instance, Yang and Ren (2019) found that L2 Motivational Self System components—specifically attitudes towards the L2 community and intended learning efforts—positively predicted pragmatic awareness. Taguchi (2014) additionally identified personality traits as predictors of L2 pragmatic competence development. More recently, Zarrinabadi et al. (2021) showed that autonomy support positively predicted language mindsets, with implications for learners’ communicative competence and WTC, while Y. Wang and Ren (2025) found that GLM predicted pragmatic awareness and WTC predicted sociopragmatic inappropriateness perception among Chinese EFL university students. Across these studies, receptive modalities have remained the exclusive focus, thereby leaving the influence of PP variables on pragmatic production largely unexplored, particularly among Chinese junior and senior EFL learners.

### 2.3. Foreign Language Perceptual Curiosity

Curiosity has long been recognized as a fundamental driver of human exploration and learning (Berlyne, 1954). Two types of curiosity were distinguished: one is epistemic curiosity, which refers to a desire to acquire knowledge and is aroused by ideas, ambiguities, and intellectual challenges that provoke a desire for understanding and insight, and the other is perceptual curiosity (PC), which “leads to increased perception of stimuli” (Berlyne, 1954, p. 180) and is evoked by novelty, complexity, and ambiguities in sensory stimulation. Henry et al. (2026) further reframed PC specifically for the foreign language learning context, developing and validating a Foreign Language Perceptual Curiosity (FLPC) Scale that conceptualizes FLPC as a situational response to specific types of target-language novelty directed toward visual inspection and discovery, rather than as a broad trait-like disposition. Since the scale asks learners to report the likelihood of responding in specified situations, however, the present study interprets its scores as retrospective self-reports of a situationally anchored tendency, rather than as direct, real-time assessments of state curiosity. The FLPC scale was designed specifically for school-aged learners, making it directly applicable to the present study, whose participants are junior and senior secondary students of comparable age.

Despite its theoretical potential, the empirical base linking curiosity to L2 learning has remained limited (Henry et al., 2026). The existing studies have predominantly regarded curiosity as a personal trait or character strength, focusing on epistemic curiosity and its associations with a range of outcomes. For instance, Mahmoodzadeh and Khajavy (2019) found that language learning curiosity was positively associated with WTC and FLE among Iranian EFL learners. Nakamura et al. (2022) identified that epistemic curiosity was fostered by positive appraisals of tasks, peer interaction, and novelty, and frequently co-occurred with positive affect and communicative desire. Nikitina et al. (2025) showed that among adolescent EFL learners, epistemic curiosity’s associations with learning outcomes varied across motivational orientations and contexts. Relatively less attention has been directed toward perceptual curiosity as a situational factor, and even less research has examined the relationship between perceptual curiosity and productive pragmatic competence in the Chinese EFL context.

FLPC is characterized by a set of features that render it particularly relevant to the study of L2 pragmatic production. Henry et al. (2026) specified that FLPC is context-dependent in its relevance, which renders FLPC itself “highly beneficial for both the learning and use of languages” (Henry et al., 2026, p. 3272). L2 pragmatic production concerns how non-native speakers acquire target-like expression in environments where authentic TL exposure is structurally limited (Ren, 2022; Taguchi & Roever, 2017). The situational conditions that FLPC is designed to capture thus map directly onto those under which L2 pragmatic production competence develops, namely limited target language contact, infrequent authentic exposure, and structurally constrained opportunities for target language use. It is therefore theoretically necessary to investigate the underlying link between FLPC and L2 pragmatic production. Accordingly, Hypothesis 1 can be formulated as follows:

**H1.**  *FLPC predicts L2 pragmatic production of requests among Chinese junior and senior school EFL learners.*

### 2.4. Foreign Language Enjoyment

Foreign Language Enjoyment (FLE) was introduced to SLA by Dewaele and MacIntyre (2014) and reconceptualized as “a complex emotion, capturing interacting dimensions of challenge and perceived ability that reflects human drive for success in the face of difficult tasks” (Dewaele & MacIntyre, 2016, p. 216). In the Chinese EFL high school context, Li et al. (2018) validated the Chinese version of the Foreign Language Enjoyment Scale among secondary students, confirming FLE as a measurable and meaningful construct for this population.

A substantial body of L2 research demonstrates that FLE is positively associated with learners’ perceived and actual L2 achievements (Dewaele & Alfawzan, 2018). Beyond its direct predictive role, FLE could have a mediating effect on grit (Wei et al., 2019), eventually influencing their actual L2 achievement. Li and Li (2023) highlighted that FLE was the strongest and most enduring predictor of L2 achievement, outweighing the negative effects of anxiety and boredom. However, the linkage between FLE and Chinese junior and senior EFL learners’ pragmatic production remains underexplored.

While FLE’s positive effects on general L2 outcomes are well-established, its function as a mediating mechanism connecting PP variables to specific learning outcomes has recently begun to receive attention. Luo (2026) found an indirect association between foreign language curiosity and learning engagement through FLE among Chinese foreign language learners, suggesting that enjoyment may be one pathway linking curiosity and engagement. Hu et al. (2022) similarly found that FLE partially mediated the relationship between growth mindset and English language performance among Chinese EFL university students. Yin et al. (2025) further showed that teacher support was indirectly associated with commitment to active learning through the sequential mediation of growth language mindset and enjoyment among Chinese college students, suggesting that situational antecedents may be linked to learning outcomes via cognitive and affective processes. However, fewer studies have examined pragmatic production as an outcome, and almost no research has yet explored whether FLE mediates the relationship between FLPC and pragmatic production ability. The following hypothesis is therefore proposed:

**H2.**  *FLE mediates the relationship between FLPC and L2 pragmatic production.*

### 2.5. Growth Language Mindset

Language mindset captures beliefs about whether one’s capacity to learn and use a foreign language is innately constrained or can be substantially developed through sustained practices (Lou & Noels, 2019). Drawing on this framework, Lou and Noels (2017) classified language mindsets into two orientations: a fixed language mindset, stating that language ability is an innate and largely unalterable quality, and a growth language mindset (GLM), reflecting the belief that language learning ability is malleable and can be enhanced through deliberate effort (Ryan & Mercer, 2012). Although language mindset encompasses both fixed and growth orientations, researchers have increasingly focused on the growth dimension in SLA (Hejazi et al., 2023). That is primarily because, methodologically, learners typically hold both orientations simultaneously, rendering their joint measurement theoretically complex (Lou & Zarrinabadi, 2022; Mercer & Ryan, 2010), and reverse-scoring fixed mindset items risks inflating measurement error (H. Wang et al., 2021). Therefore, this study also adopts GLM.

Learners possessing higher GLM demonstrate higher motivation and greater persistence in the face of failure, as well as stronger communicative competence and WTC in L2 (L. Liu & Lee, 2025; Lou & Noels, 2017; Ryan & Mercer, 2012; Zarrinabadi et al., 2021). L. Liu and Lee (2025) found that GLM mediated the ideal L2 self–WTC relationship among Chinese EFL university students, linking motivational self-images to communicative behavior. Beyond its role in communicative outcomes, GLM has also been closely associated with positive emotional states in SLA, particularly enjoyment (Hu et al., 2022; H. Wang et al., 2021; Yin et al., 2025; Zarrinabadi et al., 2022), and has shown moderating effects on the relationship between situational antecedents and learners’ affective outcomes in L2 contexts (Hejazi et al., 2023). In addition, Derakhshan et al. (2022) revealed that GLM reduced EFL learners’ boredom and thereby promoted engagement; converging with this, H. Liu et al. (2026) revealed that learners in a growth mindset profile exhibited significantly higher engagement across all dimensions than those in fixed or mixed mindset profiles. Extending these insights to the domain of L2 pragmatics, Y. Wang and Ren (2025) found that GLM positively predicted pragmatic awareness among Chinese EFL learners. However, whether GLM moderates the pathway from FLPC to FLE in the context of L2 pragmatic production remains unexplored.

Beyond its direct predictive role, GLM has consistently functioned as a moderator across both general educational and L2 learning contexts. In general educational settings, it has been found to moderate a range of learning-related outcomes (Cutumisu & Lou, 2020). In the L2 learning domain, Hejazi et al. (2023) demonstrated that GLM moderated both the teacher support–WTC relationship and the anxiety–WTC relationship, with teacher support promoting WTC and anxiety exerting a weaker inhibiting effect only among learners with medium-to-high mindset levels. This illustrates how GLM shapes learners’ sensitivity to situational learning conditions. To date, the moderating role of GLM in L2 pragmatic development has remained largely unexamined, and fewer studies have yet explored its function in relation to pragmatic production. Also, Y. Wang and Ren (2025) highlighted that productive pragmatic modalities deserve more in-depth exploration and called for future research to incorporate a broader range of psychological and emotional variables into L2 pragmatics research. The present study aims to fill this gap and proposes the following hypothesis:

**H3.**  *GLM moderates the relationship between FLPC and FLE.*

### 2.6. The Present Study

Drawing on Ren’s (2018) model of L2 pragmatic development, Control–Value Theory (Pekrun, 2006) and the Language-Mindset Meaning System (LMMS; Lou & Noels, 2019), the present study proposes a unified framework integrating FLPC, FLE, GLM, and pragmatic production. Within CVT, situational antecedents shape control-value appraisals and thereby generate achievement emotions. Accordingly, FLPC, as a situational response to target language novelty (Henry et al., 2026), may elicit FLE through learners’ positive value appraisals of novel TL stimuli. This mediating role is consistent with SLA evidence that positive emotions link situational antecedents to learning outcomes (Yin et al., 2025), and is further grounded in findings that motivational-affective variables predict L2 pragmatic awareness (Y. Wang & Ren, 2025) and in the heightened cognitive-affective demands of pragmatic production relative to comprehension (Taguchi, 2015).

**Figure 1:**
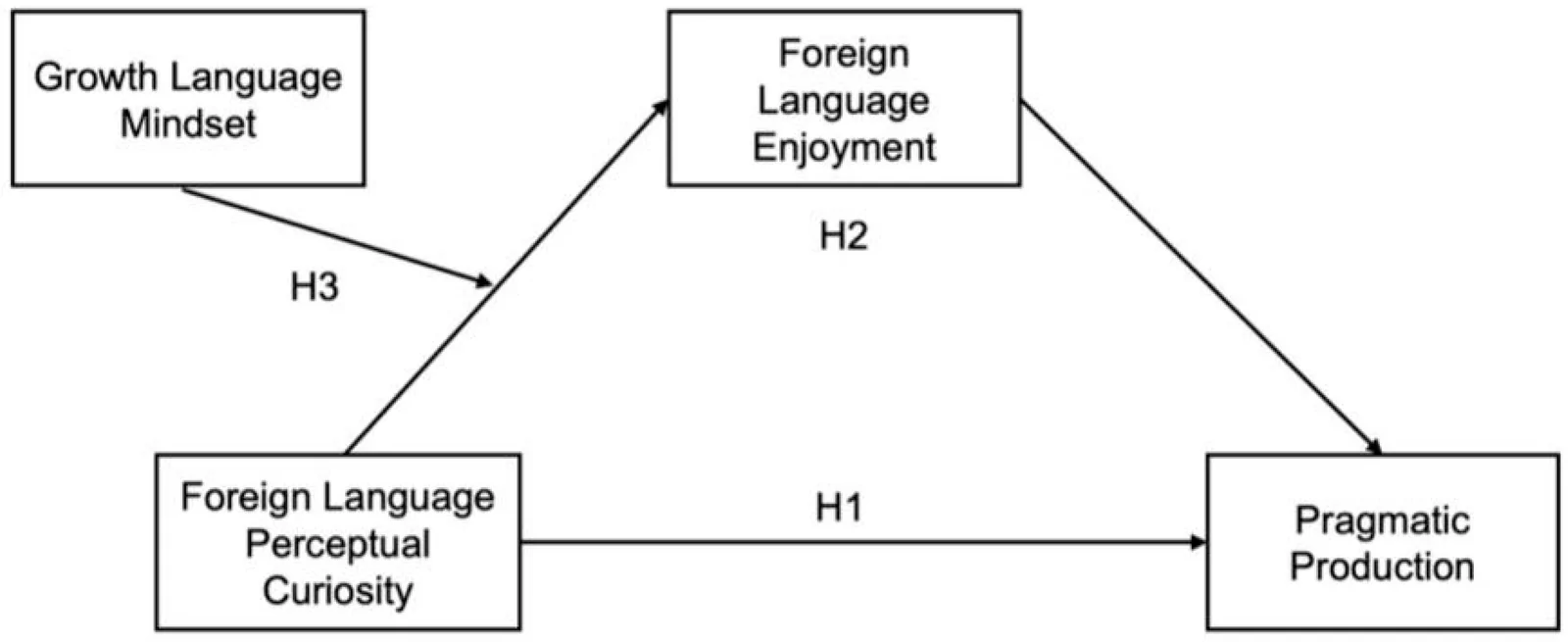
The hypothesized model.

Within the LMMS (Lou & Noels, 2019), GLM functions as an organizing construct that dynamically shapes learners’ emotional tendencies and outcome attributions in L2 learning. With evidence suggesting that it moderates the effects of situational antecedents on learning-related outcomes (Cutumisu & Lou, 2020) and on L2 WTC (Hejazi et al., 2023), GLM is proposed to moderate the FLPC→FLE pathway by shaping learners’ appraisal of subjective control and value, within the CVT framework (Pekrun, 2006), yielding a moderated mediation model. In this model (see Figure 1), FLE is specified as a mediator because the association between FLPC and pragmatic production is examined indirectly through FLE. GLM is specified as a moderator because the association between FLPC and FLE is examined at different levels of GLM. The present study, therefore, addresses the following research questions:

RQ1: To what extent does FLPC predict pragmatic production of requests among Chinese junior and senior secondary students?

RQ2: To what extent does FLE mediate the relationship between FLPC and pragmatic production?

RQ3: To what extent does GLM moderate the relationship between FLPC and FLE?

## 3. Methods

### 3.1. Participants

Participants were 381 students recruited from two secondary schools in northern China, comprising one junior secondary cohort (Grade 2; *N* = 171; *M*_age_ = 12.87, *SD* = 0.57; 52.6% female) and one senior secondary cohort (Grade 2; *N* = 210; *M*_age_ = 15.61, *SD* = 0.58; 57.6% female).

All participants were Chinese speakers enrolled in compulsory EFL instruction with no reported history of sustained immersion in English-speaking environments. Ethical approval was obtained from the relevant institutional review board. Informed consent was obtained from the participants’ parents, teachers and their schools, and assent was also obtained from the students themselves. Participation was voluntary, and participants were assured that their responses would have no bearing on academic standing or evaluations.

### 3.2. Instruments

The three psychological scales (FLE, FLPC, and GLM) were rated on a five-point Likert scale (1 = strongly disagree, 5 = strongly agree). Pragmatic production was assessed via performance-based DCT scoring, as described below.

#### 3.2.1. Pragmatic Production Test

Pragmatic production was assessed via four Discourse Completion Tasks (DCTs) targeting the speech act of requests across systematically varied power, distance, and imposition (PDI) parameters (Brown & Levinson, 1987; Ishihara & Cohen, 2022). Although written DCTs permit standardized elicitation across a large sample, they assess offline, elicited written pragmatic performance and may partly reflect knowledge of speech-act strategies, rather than directly measuring real-time spoken performance under interactional pressure (Taguchi & Roever, 2017). Responses were scored using an analytic rubric adapted from Ishihara and Cohen (2022) across three dimensions: Dimension A—tone of the request, covering overall (in)directness, (in)formality, (im)politeness of the request head act, Dimension B—choice and use of requesting strategies, including the overall amount of information provided and the selection and deployment of requesting strategies pre-specified for each item (2–3 strategies per item), and Dimension C—overall comprehensibility of the speaker’s intention in terms of appropriateness rather than accuracy, measured via word choice, grammar, and sequencing. Each dimension was scored on a 12-point scale per DCT item, yielding a maximum of 36 points per item. The overall pragmatic production score was the mean of the four item scores (range: 0–36).

Scoring was conducted by a trained research assistant following a standardized scoring protocol aligned with the analytic rubric. A second rater, an English-major research assistant who had been familiarized with the rubric through prior calibration, independently scored a randomly selected 20% subset of responses (*N* = 76) to assess inter-rater reliability. Weighted Cohen’s *κ*, calculated with quadratic weights, indicated good to excellent agreement (McHugh, 2012): overall *κ* = 0.84, Dimension A: *κ* = 0.82, Dimension B: *κ* = 0.87, and Dimension C: *κ* = 0.85.

#### 3.2.2. Foreign Language Enjoyment Questionnaire

FLE was measured using the 11-item Chinese Version of the Foreign Language Enjoyment Scale developed by Li et al. (2018), which adapted and validated Dewaele and MacIntyre’s (2014) original Foreign Language Enjoyment Scale for Chinese EFL learners. The scale captures three factors: FLE-Private (personal engagement and interest in language learning), FLE-Teacher (perceived teacher encouragement and support), and FLE-Atmosphere (positive classroom atmosphere). Sample item: “I enjoy English language learning.”

#### 3.2.3. Foreign Language Perceptual Curiosity Questionnaire

FLPC was measured using an adapted version of the Foreign Language Perceptual Curiosity Scale (Henry et al., 2026), which draws on Berlyne’s (1954) construct of perceptual curiosity to capture learners’ tendency to notice and engage with incidentally encountered target language content in digital environments. The original scale was developed with Swedish upper secondary learners; items were adapted for the Chinese EFL context by substituting target language references with English. One attention-check item was added and excluded by design prior to analysis. Confirmatory factor analysis (CFA) on the seven substantive items indicated that one item (willingness to change phone language settings to English) produced a standardized loading below 0.50 (Hair et al., 2019) and was removed, consistent with Henry et al.’s (2026) observation that this item performed weakest in their original sample. This item may have loaded weakly as changing a phone’s interface language reflects practical usability more than an immediate response to novel target-language content. Sample item: “If I come across an English video while scrolling online, I am likely to watch it.”

#### 3.2.4. Growth Language Mindset Questionnaire

Growth language mindset was assessed using items from LMI (Lou & Noels, 2017), which measures language mindsets across three dimensions: general language intelligence beliefs (GLB), second language aptitude beliefs (L2B), and age sensitivity beliefs (ASB). Only the nine growth-oriented items were administered, consistent with the study’s theoretical focus. CFA indicated that the three L2B items produced standardized loadings below 0.50 and were accordingly removed (Hair et al., 2019). This result is consistent with Lou and Noels’ (2017) original validation, where the L2B subscale returned the lowest internal consistency. The broad and absolute wording of the L2B items concerning effort-based improvement (e.g., ‘always’) may have elicited relatively uniform responses in this secondary-school sample, thereby contributing to their weak loadings. The retained six-item scale comprising three GLB items (e.g., “Anyone can change their language ability”) and three ASB items (e.g., “Regardless of age, people can learn a foreign language well if they work hard enough”) preserved the core conceptual scope of growth language mindset in the present study.

### 3.3. Data Analysis

The questionnaires were imported into SPSS 27.0 for data analysis. Preliminary analyses included descriptive statistics, normality checks, and Pearson correlations. CFA was subsequently conducted in AMOS 26 to examine the psychometric properties of each scale.

Before estimating the group-specific structural models, multigroup CFA was conducted to assess configural, metric, and scalar invariance across the junior and senior groups. Invariance was evaluated using a decrease in *CFI* of no more than 0.010 and an increase in *RMSEA* of no more than 0.015 (Chen, 2007). Structural equation modeling (SEM) was then performed for each group in AMOS 26 to test the hypothesized mediation model, using bias-corrected bootstrapping with 5000 resamples. Moderated mediation was then examined via PROCESS Model 7, specifying GLM as the moderator of the FLPC→FLE pathway. Following the CFA and SEM analyses, PROCESS Model 7 was used since it provides bootstrapped estimates of conditional indirect effects and facilitates Johnson–Neyman probing of the interaction. A sensitivity analysis incorporating gender as a covariate produced no substantive changes to any structural path estimate (all |Δ*β*| < 0.02), and gender was therefore excluded from both models.

## 4. Results

### 4.1. Reliability and Validity of Instruments Among Chinese Junior and Senior Secondary Students

The results of the CFAs and the reliability coefficients for the scales of FLE, FLPC, and GLM are presented in Table 1. All measurement models demonstrated adequate to good fit based on Kline’s (2023) guidelines that are shown in the bottom row of the table. Convergent validity was supported across all scales, with AVE values ranging from 0.52 to 0.55 (all *AVE* ≥ 0.50) and CR values ranging from 0.78 to 0.90 (all *CR* ≥ 0.70), meeting the thresholds recommended by Hair et al. (2019). Internal consistency was satisfactory for all scales, with *Cronbach’s α* ranging from 0.838 to 0.891 (all *α* > 0.70). The measurement model showed acceptable configural fit, *χ*^2^*/df* = 1.603, *CFI* = 0.942, *TLI* = 0.932, *RMSEA* = 0.056, and *SRMR* = 0.054. Changes in model fit after constraining factor loadings (Δ*CFI* = −0.003, Δ*RMSEA* < 0.001) and item intercepts (Δ*CFI* = −0.009, Δ*RMSEA* = 0.002) supported metric and scalar invariance, respectively.

Descriptive statistics and Pearson correlations for both groups are presented in Table 2. All variables met the normality criteria recommended by Kline (2023) in both sample groups. Although Table 2 shows a marked descriptive difference in pragmatic production, with the senior group scoring higher (*M* = 27.07, *SD* = 5.91) than the junior group (*M* = 17.97, *SD* = 10.44), this cross-sectional comparison of independent cohorts should not be interpreted as evidence of within-learner developmental change. In both groups, the three latent variables—FLPC, FLE, and GLM—were positively correlated with pragmatic production (all *p* < 0.05), with FLE exhibiting the strongest association in the junior (*r* = 0.307, *p* < 0.001) and senior groups (*r* = 0.303, *p* < 0.001). The three psychological variables were also positively and significantly intercorrelated (all *p* < 0.001), with the FLE–GLM correlation being notably strong in both groups (*r* = 0.561 for the junior group; *r* = 0.532 for the senior one).

### 4.2. The Results of the Research Questions

#### 4.2.1. RQ1: Foreign Language Perceptual Curiosity and Pragmatic Production

**Figure 2:**
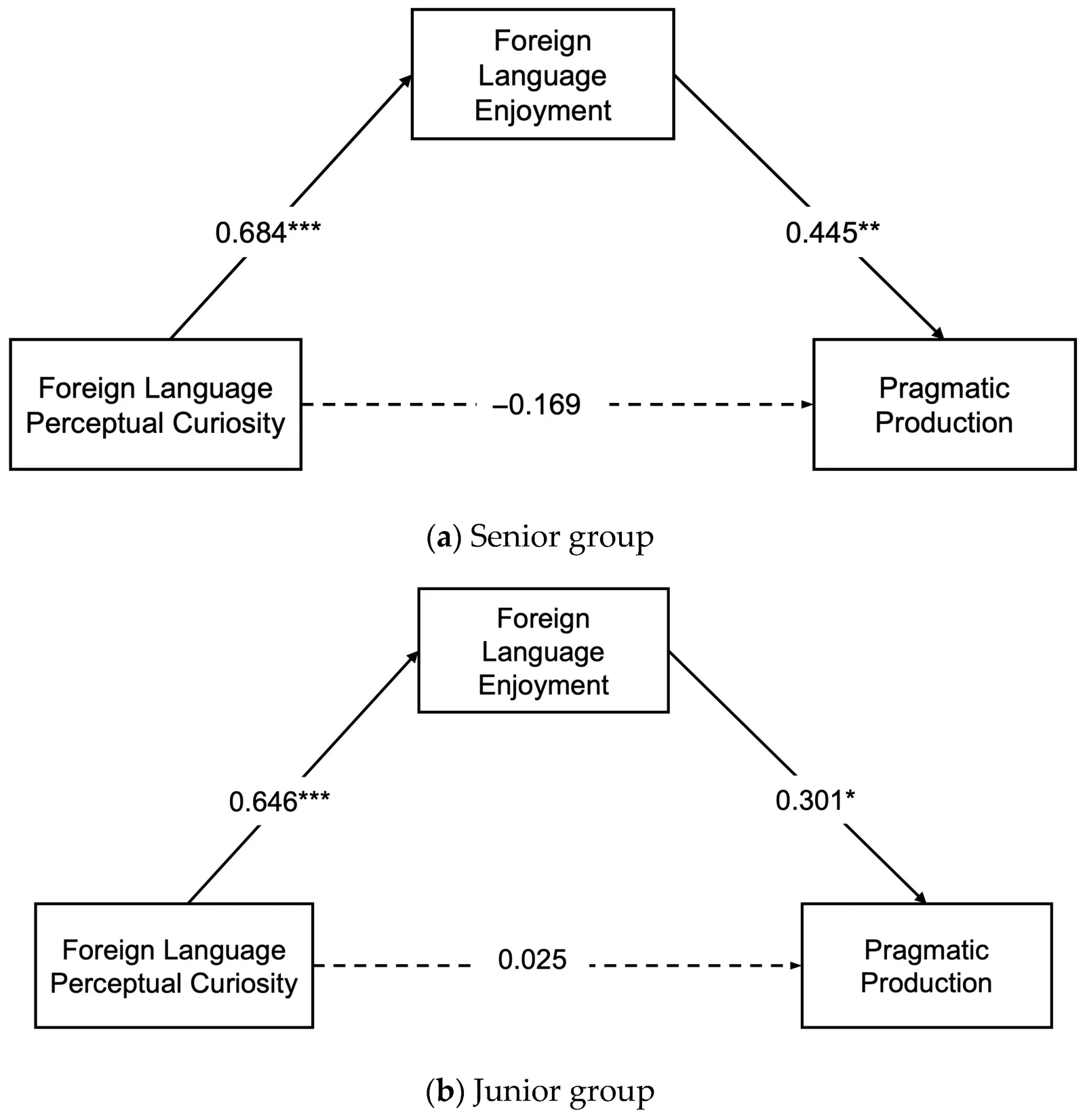
Structural mediation models for the senior and junior groups. ***Note.*** * *p* < 0.05. ** *p* < 0.01. *** *p* < 0.001. Solid arrows indicate statistically significant paths, whereas the dashed arrow indicates the non-significant path.

Table 3 presents the direct and indirect effects and *95% CI* for the structural models (see also Figure 2). Regarding the RQ1, the direct effect of FLPC on pragmatic production was non-significant in the junior group (*β* = 0.025, *p* = 0.838) and the senior group (*β* = −0.169, *p* = 0.221), indicating that FLPC did not independently predict pragmatic production in either group. Therefore, H1 was not supported.

#### 4.2.2. RQ2: The Mediating Role of Foreign Language Enjoyment

Regarding RQ2, the mediating paths of FLE for both groups are presented in Figure 2. The SEM models demonstrated acceptable fit for the senior group (*χ*^2^/*df* = 1.545, *CFI* = 0.957, *TLI* = 0.950, *RMSEA* = 0.051, *SRMR* = 0.063) and the junior group (*χ*^2^/*df* = 1.667, *CFI* = 0.946, *TLI* = 0.937, *RMSEA* = 0.063, *SRMR* = 0.075). FLPC predicted FLE in both the junior group (*β* = 0.646, *p* < 0.001) and the senior group (*β* = 0.684, *p* < 0.001). FLE in turn positively predicted pragmatic production in both groups (junior: *β* = 0.301, *p* = 0.021; senior: *β* = 0.445, *p* = 0.001). Table 3 displays that the indirect effect of FLPC on pragmatic production via FLE was significant in both the junior group (*β* = 0.194, *p* = 0.018) and the senior group (*β* = 0.304, *p* = 0.001), with both direct paths to pragmatic production remaining non-significant—consistent with full mediation in both groups (H2 supported).

#### 4.2.3. RQ3: The Moderating Role of Growth Language Mindset

The results of the moderated mediation analysis (PROCESS Model 7) are presented in Table 4. As for RQ3, evidence for the moderating role of GLM emerged in the senior group but not in the junior group.

**Figure 3:**
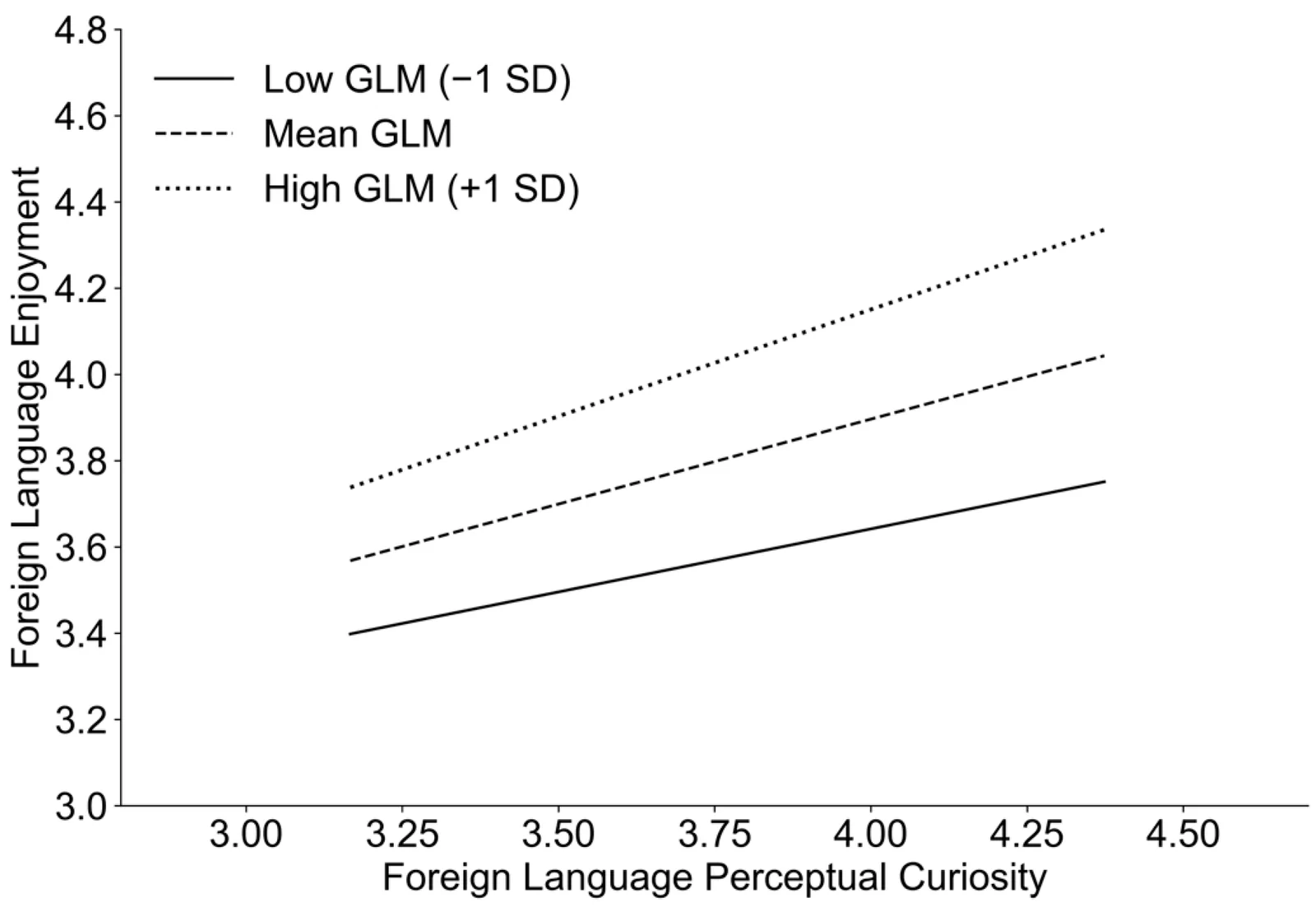
The moderating effect of GLM on the relationship between FLPC and FLE (senior group).

**Figure 4:**
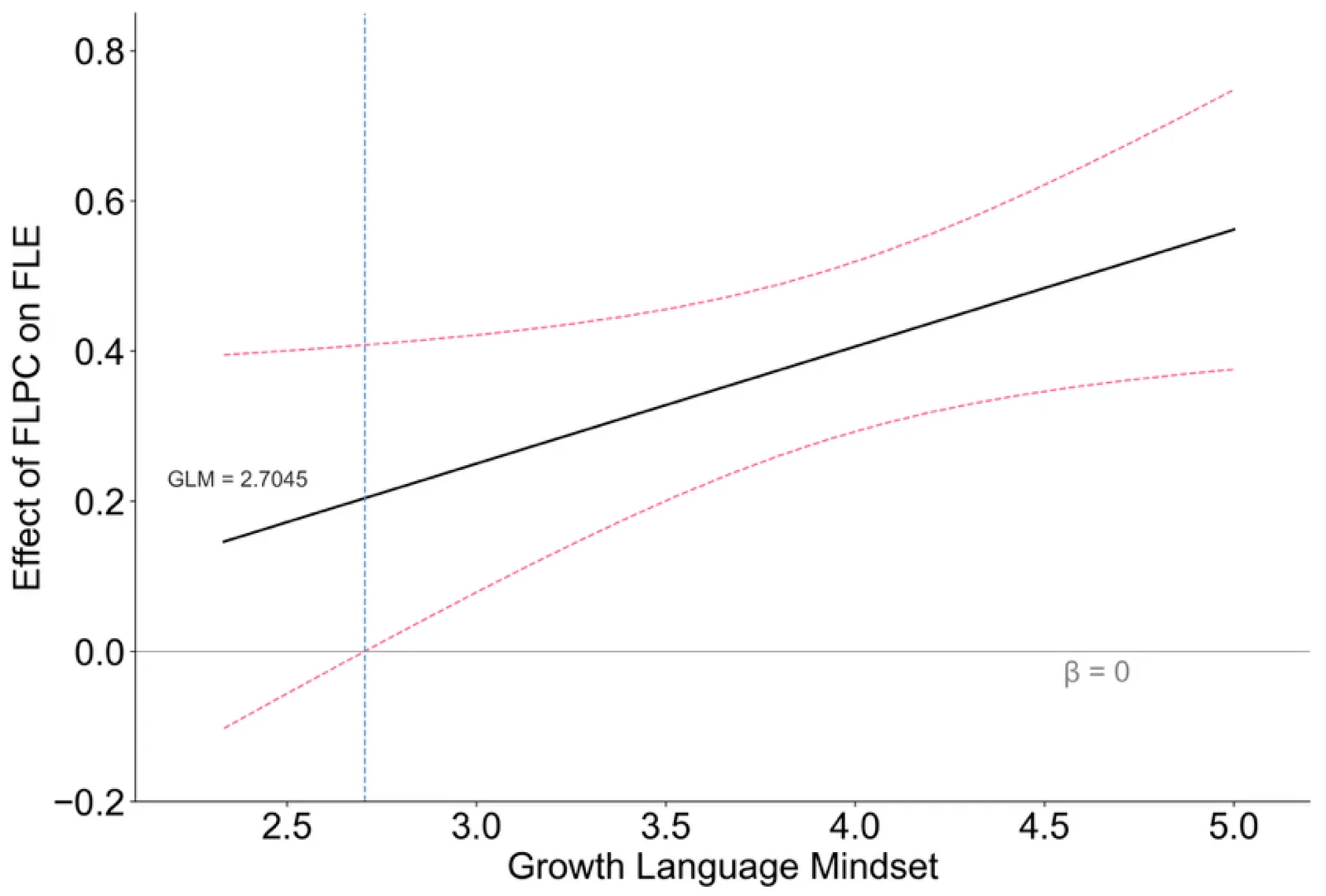
Johnson–Neyman plot of the moderating effect of GLM on the FLPC→FLE pathway (senior group). Red dashed lines indicate the 95% CI, and the blue dashed line marks the Johnson–Neyman point (GLM = 2.7045).

In the senior group, the FLPC × GLM interaction significantly predicted FLE (*β* = 0.156, *p* = 0.028). The index of moderated mediation was 0.472 (*SE* = 0.255, *95% CI* = [0.009, 1.010]), excluding zero. As shown in Figure 3, simple slope analysis revealed that the positive effect of FLPC on FLE strengthened as GLM increased from low (−1 *SD*) to high (+1 *SD*), with all three conditional indirect effects excluding zero. Johnson–Neyman analysis (see Figure 4) further indicated that the moderating effect became significant when GLM exceeded 2.7045, a threshold surpassed by 94.76% of the senior participants. Given the modest interaction and the confidence interval close to zero, this result should be interpreted cautiously. It indicates a conditional association within the senior sample rather than establishing a developmental difference from the junior group. In the junior group, the interaction term was non-significant (*β* = −0.080, *p* = 0.312), and the index of moderated mediation contained zero. H3 was therefore partly supported, that is, the hypothesized interaction was significant in the senior group but not in the junior group.

## 5. Discussion

The present study examined whether foreign language perceptual curiosity (FLPC) is associated with L2 pragmatic production of requests among Chinese EFL junior and senior learners through a moderated mediation model in which foreign language enjoyment (FLE) serves as a mediator and growth language mindset (GLM) as a moderator of the FLPC→FLE pathway. The findings indicate that FLPC shows no significant direct association with pragmatic production in either group; instead, a statistical mediation pattern involving FLE was observed in both groups, with GLM significantly moderating the FLPC→FLE association only among senior secondary students. Given the cross-sectional design, these paths are interpreted as statistical associations within the tested model. These findings highlight the role of affective and belief-related variables in the statistical association between situational curiosity and productive pragmatic performance in instructed Chinese EFL settings, suggesting that L2 positive emotions and growth-oriented language learning beliefs are relevant within the tested model rather than FLPC acting in isolation.

Regarding the first research question, the non-significant direct effect of FLPC on pragmatic production suggests that situational curiosity is not directly associated with pragmatic production in the tested model. This finding aligns with Control–Value Theory (CVT) (Pekrun, 2006), which posits that FLPC functions as a distal antecedent that shapes achievement outcomes (viz. pragmatic production) primarily through emotion-based appraisals rather than direct pathways. That is, FLPC does not itself generate the sustained attentional and behavioral resources required for L2 pragmatic production of requests—a face-threatening act demanding real-time management of sociopragmatic variables such as power, distance, and imposition (Brown & Levinson, 1987), alongside the selection of politeness strategies (Taguchi & Roever, 2017). The situationally anchored tendency measured by FLPC (Henry et al., 2026) may be associated with attention to pragmatic input but does not by itself provide the sustained engagement or regulatory resources necessary for successful pragmatic output. At the same time, pragmatic production imposes higher cognitive-affective demands than receptive pragmatic tasks (Taguchi, 2015), suggesting that such a situationally anchored tendency may be insufficient without more enduring emotional orientations. This interpretation aligns with broader SLA evidence showcasing that curiosity-related constructs—particularly epistemic curiosity—tend to influence L2 outcomes indirectly via motivational or affective channels (Luo, 2026; Mahmoodzadeh & Khajavy, 2019). The present study extends this structural pattern to perceptual curiosity and to the specific domain of L2 pragmatic production, thereby refining our understanding of how situational affect operates in output-oriented pragmatic competence.

As for the second research question, the mediation analysis identifies a significant indirect association between FLPC and productive pragmatic performance through FLE, further including tone, strategy deployment, and intention comprehensibility of requests. The strong FLPC→FLE pathway corroborates recent evidence linking foreign language curiosity to enjoyment and learning engagement (Luo, 2026). More importantly, the significant FLE→pragmatic production pathway provides new empirical evidence that L2 enjoyment is positively associated with not only general L2 achievements (Dewaele & Alfawzan, 2018) but also the more cognitively demanding domain of productive pragmatic performance compared to receptive pragmatic comprehension (Y. Wang & Ren, 2024). Within CVT, positive activity emotions arise from high value appraisals of novel stimuli and subsequently promote flexible cognitive repertoires, which are essential for selecting pragmatically appropriate request strategies and managing interpersonal meaning. The full mediation pattern observed in both groups indicates a statistical indirect association between FLPC and pragmatic performance through FLE within the tested model. This finding converges with studies demonstrating that positive emotions mediate the effects of motivational and contextual variables on L2 learning outcomes (e.g., Hu et al., 2022; Yin et al., 2025), while extending this mechanism specifically to L2 pragmatic production—a domain largely overlooked in prior positive psychology research.

With regard to the third question, GLM was associated with a modest conditional pattern in the FLPC→FLE pathway among Chinese senior high school learners, whereas this interaction was not statistically significant among junior learners. This resonates with Hejazi et al. (2023), who similarly found that GLM moderated a situational antecedent’s effect on WTC only at medium-to-high mindset levels. This pattern empirically supports the Language-Mindset Meaning System (Lou & Noels, 2019), in which growth beliefs function as an organizing construct that sensitizes learners to situational antecedents under conditions of greater academic pressure. That is, learners with stronger growth mindsets are more likely to interpret novel or challenging pragmatic input as opportunities for development, thereby amplifying the emotional benefits of curiosity. The absence of this moderating effect in the junior group suggests that GLM may differ across the two samples. This group-specific pattern may be related to learners’ accumulated experience and adaptive capacity. On the one hand, Chinese EFL senior students, who face greater academic pressure and more complex linguistic examination demands, may rely more heavily on mindset-based self-regulation to sustain positive engagement. This interpretation is compatible with Zarrinabadi et al. (2022), who identified adaptability as a mechanism linking growth mindset to positive emotions, consistent with Lou and Noels’ (2019) observation that mindset effects tend to magnify under more challenging transitional conditions. On the other hand, Chinese junior learners, facing comparatively lower stakes, may derive enjoyment from FLPC regardless of mindset orientation. The finding thus qualifies earlier claims of mindset effects (Y. Wang & Ren, 2025; Zarrinabadi et al., 2021) by demonstrating that the moderating role of GLM may not be uniform across educational stages.

Taken together, these findings offer several theoretical contributions. First, by identifying an indirect association between FLPC and pragmatic production via FLE, the present study extends Control–Value Theory (Pekrun, 2006) into the domain of L2 pragmatic production while simultaneously advancing Ren’s (2018) model of factors influencing L2 pragmatic competence; specifically, it integrates FLPC as a distinct variable and identifies an indirect association via FLE and a group-specific moderation pattern through which situational curiosity is associated with productive rather than receptive pragmatic competence. Second, by incorporating FLPC as a situational construct, the study advances existing research on curiosity in SLA, which has predominantly focused on trait-level epistemic curiosity. Third, the observed school-stage-specific pattern in the moderating role of GLM refines the Language-Mindset Meaning System by suggesting possible variability across educational contexts. These contributions respond directly to recent calls for integrating positive psychological variables into L2 pragmatics research (Y. Wang & Ren, 2024, 2025) and for moving beyond receptive modalities toward production-based pragmatic outcomes (Ren, 2022; Taguchi & Roever, 2017).

From a pedagogical perspective, the observed indirect association via FLE suggests that pragmatic instruction may benefit from context-rich pragmatic tasks and explicit pragmatic instruction (Ren et al., 2023). The group-specific moderation pattern was modest and should be replicated before it informs differential intervention. If replicated, mindset-oriented feedback that frames errors as learning opportunities may be particularly relevant for senior learners (Lou & Noels, 2016), whereas supportive classroom environments may be especially relevant for junior learners.

## 6. Conclusions

The present study shows that FLPC is indirectly associated with L2 request production through FLE among Chinese senior and junior secondary learners, with a significant interaction between FLPC and GLM observed only among senior learners, thereby offering the empirical test of a moderated mediation model linking perceptual curiosity to productive pragmatics. By integrating situational, emotional, and belief-based positive psychological variables into a unified framework, the findings further provide a more comprehensive account of how individual differences interact to shape productive pragmatic competence, particularly in Chinese secondary EFL contexts.

Despite these contributions, several limitations should be acknowledged. First, the cross-sectional design precludes causal inference regarding the relationships among FLPC, FLE, GLM, and pragmatic production. Longitudinal or experimental designs would be necessary to establish temporal and causal ordering. Second, the sample was drawn from two secondary schools, which may limit the generalizability of the findings to other educational settings or cultural backgrounds. Third, pragmatic production was measured via written discourse completion tasks (DCTs), which, while widely used, may not fully capture the dynamic and interactive nature of real-time pragmatic performance. Finally, all psychological variables were assessed through self-report measures, which may be subject to social desirability or response bias. Future research could address these limitations by employing longitudinal designs, multi-site sampling, and mixed-method assessments of pragmatic output to examine whether these mechanisms generalize across proficiency levels or instructional settings, ultimately refining both theoretical models of L2 pragmatics and evidence-based pedagogical practices.

## Figures and Tables

**Table 1:** CFA results and reliability indices of the measurement instruments.

Variable	*CMIN/DF*	*CFI*	*TLI*	*RMSEA*	*SRMR*	*Cronbach’s α*	*CR*	*AVE*
FLE	2.749	0.972	0.962	0.068	0.043	0.891	0.90	0.55
FLPC	2.205	0.979	0.961	0.056	0.035	0.850	0.78	0.52
GLM	2.923	0.984	0.969	0.071	0.027	0.838	0.85	0.53
Acceptable Value	<3	>0.90	>0.90	<0.08	<0.08	>0.70	≥0.70	≥0.50

***Note***. FLE = foreign language enjoyment; FLPC = foreign language perceptual curiosity; GLM = growth language mindset.

**Table 2:** Descriptive statistics and Pearson correlations.

Variable	Junior Group				Senior Group			
	1	2	3	4	1	2	3	4
1. FLE	–				–			
2. FLPC	0.568 ***	–			0.543 ***	–		
3. GLM	0.561 ***	0.364 ***	–		0.532 ***	0.390 ***	–	
4. PPro	0.307 ***	0.238 **	0.191 *	–	0.303 ***	0.156 *	0.269 ***	–
*Mean*	4.01	3.88	3.85	17.97	3.85	3.81	3.92	27.07
*SD*	0.63	0.73	0.63	10.44	0.60	0.60	0.65	5.91
*Skewness*	−0.289	−0.731	−0.079	−0.306	−0.017	−0.230	−0.143	−0.793
*Kurtosis*	−0.457	0.762	−0.497	−0.922	0.235	−0.284	−0.276	0.186

***Note***. PPro = pragmatic production; * *p* < 0.05. ** *p* < 0.01, *** *p* < 0.001.

**Table 3:** Direct and indirect effects and 95% CI for the mediated models.

Model Pathways	Junior Group			Senior Group			Hypothesis Testing
	*β*	*p*	*95% CI*	*β*	*p*	*95% CI*	
*Direct effect*							
FLPC → FLE	0.646	<0.001	[0.374, 0.829]	0.684	<0.001	[0.530, 0.808]	
FLPC→PPro	0.025	0.838	[−0.360, 0.312]	−0.169	0.221	[−0.480, 0.107]	H1 ^n.s.^
FLE→PPro	0.301	0.021	[0.019, 0.655]	0.445	0.001	[0.192, 0.724]	
*Indirect effect*							
FLPC → FLE →PPro	0.194	0.018	[0.032, 0.583]	0.304	0.001	[0.140, 0.569]	H2 * for junior; H2 ** for senior

***Note.*** ^n.s.^ = not statistically significant; * *p* < 0.05. ** *p* < 0.01.

**Table 4:** Conditional indirect effects of FLPC on pragmatic production via FLE at different levels of growth language mindset.

Independent Variable	Moderator (GLM)	*Effect*	*SE*	*LL 95% CI*	*UL 95% CI*	Hypothesis Testing
**Junior group**						
FLPC	−1 *SD* (3.220)	1.725	0.759	0.424	3.357	
	*M* (3.852)	1.515	0.642	0.403	2.887	
	+1 *SD* (4.484)	1.305	0.607	0.327	2.682	
*Index of moderated mediation*		−0.332	0.389	−1.138	0.401	H3 ^n.s.^ for junior
**Senior group**						
FLPC	−1 *SD* (3.271)	0.885	0.339	0.312	1.651	
	*M* (3.921)	1.192	0.364	0.522	1.951	
	+1 *SD* (4.572)	1.499	0.454	0.668	2.466	
*Index of moderated mediation*		0.472	0.255	0.009	1.010	H3 * for senior

***Note***. FLPC = foreign language perceptual curiosity; GLM = growth language mindset; ^n.s.^ = not statistically significant. Effects are unstandardized conditional indirect effects estimated using PROCESS Model 7. * *p* < 0.05.

## Data Availability

The data presented in this study are available on request from the corresponding author due to ethical restrictions and privacy considerations.
